# Zinc transporter ZIP10 supports zinc homeostasis and myoglobin biosynthesis in differentiating C2C12 myoblasts

**DOI:** 10.3389/fcell.2025.1691317

**Published:** 2025-11-19

**Authors:** Geonu Shim, Min Ji Kim, Moon-Suhn Ryu

**Affiliations:** Department of Food and Nutrition, College of Human Ecology, Yonsei University, Seoul, Republic of Korea

**Keywords:** zinc deficiency, SLC39A10, myogenesis, gene silencing, nitric oxide metabolism

## Abstract

**Background:**

Zinc is an essential micronutrient required for diverse cellular processes, including skeletal muscle development and regeneration. Although skeletal muscle contains a large proportion of total body zinc, the mechanisms that maintain zinc homeostasis during myoblast differentiation, particularly under zinc-limited conditions, remain poorly understood.

**Methods:**

We investigated ZIP10 (SLC39A10) as the primary zinc importer responsible for maintaining intracellular zinc homeostasis under fluctuating zinc conditions during C2C12 myoblast differentiation. Zinc levels were modulated using zinc chelation or supplementation, and ZIP10 expression was repressed by gene silencing. Molecular and functional signatures of myogenesis were assessed through gene and protein expression analyses and nitric oxide (NO) metabolite profiling.

**Result:**

Zinc deficiency markedly reduced the expression of the muscle-specific transcription factor *Myog*, as well as *Mb*. Among the 14 *Zip* and 10 *ZnT* genes profiled, *Zip10* showed the largest fold increase in response to zinc depletion. *Zip10* knockdown had minimal effects under zinc-sufficient conditions but further decreased *Mb* expression in zinc-deficient myoblasts without altering viability or major myogenic transcription factors. Notably, while zinc deficiency suppressed *Mb* expression, total cellular heme content remained unchanged, suggesting a zinc-dependent regulation of *Mb* biosynthesis independent of heme availability. Zinc deficiency also altered NO metabolism, as reflected by elevated nitrite levels and a reduced nitrate-to-nitrite ratio, indicating impaired Mb-mediated NO detoxification. Zinc addition elevated *Mb* expression and improved cell viability, with effects observed across both early and late differentiation phases.

**Conclusion:**

These findings identify ZIP10 as an important zinc importer that supports intracellular zinc supply and *Mb* expression during myogenic differentiation, offering new insight into the nutritional regulation of muscle physiology by zinc.

## 1 Introduction

Zinc (Zn) is an essential trace element required for fundamental cellular processes, including nucleic acid synthesis, enzymatic catalysis, transcriptional regulation, and intracellular signaling (MacDonald, 2000; Rink and Gabriel, 2000; Kambe et al., 2015; Marreiro et al., 2017). Skeletal muscle contains nearly 60% of the body’s total zinc (Jackson, 1989). During myogenesis, proliferating and differentiating myoblasts experience substantial metabolic and biosynthetic demands, making zinc availability critical for proper progression of muscle fiber formation (Reddy et al., 2022). In contrast to iron, zinc is not stored in a readily mobilizable pool. Instead, its intracellular levels rely on continuous dietary supply, association with metallothioneins (MTs), and the coordinated action of zinc transporters controlling uptake, sequestration, and efflux (Rink and Gabriel, 2000; Colvin et al., 2010). Consequently, zinc deficiency (which remains widespread globally) can impair muscle development, repair, and function (Park et al., 1986; Van Loan et al., 1999; Jinno et al., 2014). Despite this, the molecular mechanisms that maintain zinc homeostasis in differentiating myoblasts, particularly under zinc-limited conditions, are not well understood.

Notably, intracellular zinc content has been reported to rise during myogenic differentiation, reflecting the increased requirement for zinc as myoblasts progress toward myotube (Paskavitz et al., 2018). To address this increased demand, it is essential to consider the zinc transport systems that regulate intracellular flux. Zinc flux into and out of the cytosol is mediated by two transporter families: ZIP (SLC39A), which facilitates zinc import from the extracellular space or intracellular stores, and ZnT (SLC30A), which promotes efflux or sequestration into organelles (Colvin et al., 2010; Kambe et al., 2015). ZIP transporters are expressed in skeletal muscle, and their abundance is dynamically regulated during differentiation (Myers et al., 2013; Paskavitz et al., 2018). ZIP7, located in the endoplasmic reticulum and Golgi apparatus, influences insulin signaling and glucose metabolism in myoblasts (Myers et al., 2013; Mnatsakanyan et al., 2018), while loss-of-function mutations in ZIP13 result in connective tissue defects and impaired myogenesis in human cells (Bin et al., 2014; Shoji et al., 2024). These examples highlight the importance of finely tuned zinc transport for muscle cell development. However, the transporter primarily responsible for sustaining intracellular zinc availability under zinc-deficient conditions during myogenic differentiation has not been clearly defined.

Zinc may influence the oxygen-handling capacity in muscle through its role in heme biosynthesis. In this pathway, zinc serves as a cofactor for δ-aminolevulinate dehydratase (ALAD), a key enzyme driving porphyrin synthesis and ultimately heme production (Jaffe, 2016). Heme is subsequently incorporated into numerous metalloproteins, including myoglobin (Mb), which is abundant in oxidative muscle fibers (Ordway and Garry, 2004; Kamga et al., 2012). Mb facilitates oxygen storage and diffusion, modulates nitric oxide (NO) metabolism, and provides protection against oxidative and nitrosative stress in muscle tissue (Ordway and Garry, 2004). Evidence from erythroid cells shows that zinc deficiency impairs heme biosynthesis (Kim et al., 2023). However, whether insufficient zinc availability similarly disrupts Mb expression and function in skeletal muscle remains unclear.

The expression of MTs and zinc transporters is regulated by the metal-responsive transcription factor-1 (MTF-1), which coordinates cellular adaptation to zinc status. Under zinc-replete conditions, MTF-1 induces genes such as *Mt1* and *ZnT1* to enhance zinc buffering and efflux, while repressing *Zip10* transcription by interfering with RNA polymerase II elongation (Lichten et al., 2011). When extracellular zinc becomes limited, this repression is relieved, and ZIP10 has been proposed to function as part of a compensatory response to low zinc availability, increasing zinc influx when cellular demand exceeds supply (Lichten et al., 2011). Considering the high zinc requirement of differentiating myoblasts and the potential sensitivity of heme protein biosynthesis to zinc status, ZIP10 may play a critical, yet uncharacterized, role in sustaining zinc-dependent processes such as Mb expression during myogenesis.

Here, we investigated the role of ZIP10 in regulating zinc homeostasis during myogenic differentiation of C2C12 myoblasts under zinc-deficient conditions. We hypothesized that the induction of ZIP10 represents an adaptive mechanism to sustain intracellular zinc supply when demand exceeds extracellular availability. Our findings demonstrate that ZIP10 is required to maintain Mb expression under zinc restriction, revealing a critical contribution of this transporter to zinc-dependent processes during muscle differentiation.

## 2 Materials and methods

### 2.1 Cell culture, differentiation, and chemical treatments

The C2C12 cell line is a widely used model of immortalized myoblasts, initially established from the thigh muscle of mice (Yaffe and Saxel, 1977), and undergoes myogenic differentiation under low serum conditions (Blau et al., 1983; Andres and Walsh, 1996). C2C12 myoblasts were cultured in growth medium (GM), composed of high-glucose DMEM (HyClone, SH30243.01) supplemented with 10% fetal bovine serum and 100 U/mL penicillin-streptomycin (HyClone, SV30010) at 37 °C in 5% CO2. Cells were maintained at <70% confluency and kept at a low passage number. To induce differentiation, cells were cultured until they reached 70%–90% confluency, after which the GM was replaced with differentiation medium (DM) containing 2% heat-inactivated horse serum (Gibco, 26050088) in place of 10% FBS. The DM was refreshed every 2 days. Zinc depletion and supplementation were induced by treating cells with a cell-impermeable zinc chelator, diethylenetriamine pentaacetate (DTPA; Sigma-Aldrich, D6518) and zinc chloride (ZnCl_2_; Sigma-Aldrich, Z0152), respectively. Final concentrations of each chemical were as specified in the Results section and detailed in the corresponding figure legends.

### 2.2 siRNA-based gene knockdown

For gene silencing, cells were transfected using HiPerFect Transfection Reagent (QIAGEN, 301705) according to the manufacturer’s instructions. AccuTarget Negative Control siRNA (Bioneer, SN-1003) served as the negative control, and the Silencer Select siRNA targeting *Slc39a10* (Thermo Fisher Scientific, 4390771) was used for *Zip10* knockdown. Briefly, each siRNA and HiPerFect reagent were separately diluted in DMEM and mixed at 1:1 ratio. Following a 10-min incubation at room temperature to allow complex formation, the mixture was added to the cells, resulting in a final siRNA concentration of 60 nM.

### 2.3 RNA isolation, reverse transcription, and quantitative PCR (qPCR)

Cells were rinsed with ice-cold PBS, detached using a scraper, and pelleted by centrifugation at 600 × g for 5 min at 4 °C. Total RNA was extracted from cells using TRIzol reagent (Sigma-Aldrich) following the manufacturer’s instructions. Equal amounts of extracted RNA were used for reverse transcription to synthesize cDNA using PrimeScript RT Master Mix (Perfect Real Time) (TAKARA, RR036A), according to the manufacturer’s protocol. Prior to qPCR, cDNA samples were diluted 10-fold in nuclease-free water. qPCR was performed using TB Green Fast qPCR Mix (TAKARA, RR430A) on a CFX Duet Real-Time PCR System (Bio-Rad). Relative mRNA expression was quantified using the 2-ΔΔCq method, with fold changes normalized to *Gapdh* or *Actb* as housekeeping genes. Primer sequences for each gene are listed in Supplementary Table S1.

### 2.4 Protein extraction and Western blot analysis

Cell pellets were lysed in RIPA buffer supplemented with Halt Protease Inhibitor Cocktail (Thermo Fisher Scientific, 78430). Lysates were centrifuged at 12,000 × g for 10 min at 4 °C, and the resulting supernatants were collected. Equal amounts of protein were mixed with 4X Laemmli Sample Buffer (1610747, Bio-Rad) containing 10% 2-mercaptoethanol (Bio-Rad, 1610719) and incubated on a rotator at 4 °C for 30 min for denaturation. Denatured proteins were separated by PAGE using 4%–20% Mini-PROTEAN TGX Stain-Free Protein Gels (Bio-Rad, 4568096) in Tris/Glycine/SDS Electrophoresis buffer (Bio-Rad, 1610772). The separated proteins were transferred onto a 0.2 μm nitrocellulose membrane (Bio-Rad, 1704270) using the Trans-Blot Turbo system (Bio-Rad). To minimize non-specific binding, the membrane was blocked with 5% (w/v) milk for 1 h at room temperature. Membranes were then incubated at 4 °C with primary antibodies: rabbit anti-Mb (1:1,000; 25919, Cell Signaling Technology), rabbit anti-ZIP10 (1:2000; PA5-21064, Thermo Fisher Scientific), mouse anti-MT1 (1:1,000; MA1-25479, Invitrogen), and anti-GAPDH (1:5,000; 12004167, Bio-Rad). After primary incubation, proteins were probed with secondary antibodies (680 or 800 nm; 1:20,000, Li-Cor) for 1 h at room temperature. Relative protein expression was visualized using the Odyssey XF Imaging System (Li-Cor).

### 2.5 Giemsa stain and fusion index measurement

Cell morphology was visualized by Giemsa staining. After removing the culture medium, cells were washed three times with ice-cold PBS and fixed with methyl alcohol (DAEJUNG, 5,558–4,105) for 10 min at room temperature. The cells were then air-dried for 15 min and stained with Giemsa solution (DAEJUNG, 4,060–1,440) for 10 min. Excess stains were removed by washing the cells with distilled water, followed by air-drying for at least 30 min. To quantify myotube formation, the fusion index was calculated as the percentage of nuclei within multinucleated myotubes relative to the total number of nuclei.

### 2.6 Measuring cell viability

Relative cell viability was assessed using the Cell Counting Kit-8 (CCK-8; Dojindo, CK04-11), which is based on the reduction of the tetrazolium salt WST-8 by cellular dehydrogenase. The medium was removed from each well of a 96-well plate. A mixture of fresh medium and CCK-8 reagent (10:1 ratio) was added to each well. The plate was incubated at 37 °C with 5% CO_2_ for 2 h, and absorbance was measured at 450 nm.

### 2.7 Heme assay

Cellular heme content was quantified using the QuantiChrom Heme Assay Kit (BioAssay Systems, DIHM-250) which is based on colorimetric detection. Cell pellets were lysed by incubation with the assay reagent at room temperature for 5 min, followed by using a QIAshredder (QIAGEN, 79654) at 12,000 × g for 2 min at room temperature. Absorbance was measured at 400 nm, and heme levels were normalized to total protein content.

### 2.8 Nitric oxide assay

Cellular NO metabolites were quantified using the Total Nitric Oxide and Nitrate/Nitrite Parameter Assay Kit (R&D Systems, KGE001). Culture media were centrifuged at 1,500 × g for 10 min at 4 °C, and the resulting supernatants were used for analysis. Nitrite (NO_2_^−^) concentration was measured directly, and total NO was determined by converting all nitrate (NO_3_^−^) to NO_2_^−^ within the same sample. NO_3_^−^ levels were then calculated by subtracting the measured NO_2_^−^ from the total NO concentration. For evaluation of NO metabolism, cells were treated with 50 μM sodium nitroferricyanide (III) dihydrate (SNP; Sigma-Aldrich 228710) as an NO donor for 6 h.

### 2.9 Statistical analysis

All quantitative data are presented as the mean ± standard deviation (SD) from at least three biological replicates or independent experiments. Statistical comparisons between two groups were performed using a two-tailed Student’s t-test. For comparisons among multiple groups, one-way ANOVA followed by Tukey’s honestly significant difference (HSD) *post hoc* test was applied. Repeated-measures ANOVA with Bonferroni’s *post hoc* correction was used for datasets collected across different time points. For experiments involving two independent variables, two-way ANOVA was used to assess the main effects and their interaction. A *P*-value <0.05 was considered statistically significant. All analyses were performed using SPSS software (IBM, Version 27), except for two-way ANOVA, which was conducted using GraphPad Prism (Version 10).

## 3 Results

### 3.1 Zinc depletion induced by high-dose DTPA disrupts C2C12 differentiation and viability

**FIGURE 1:**
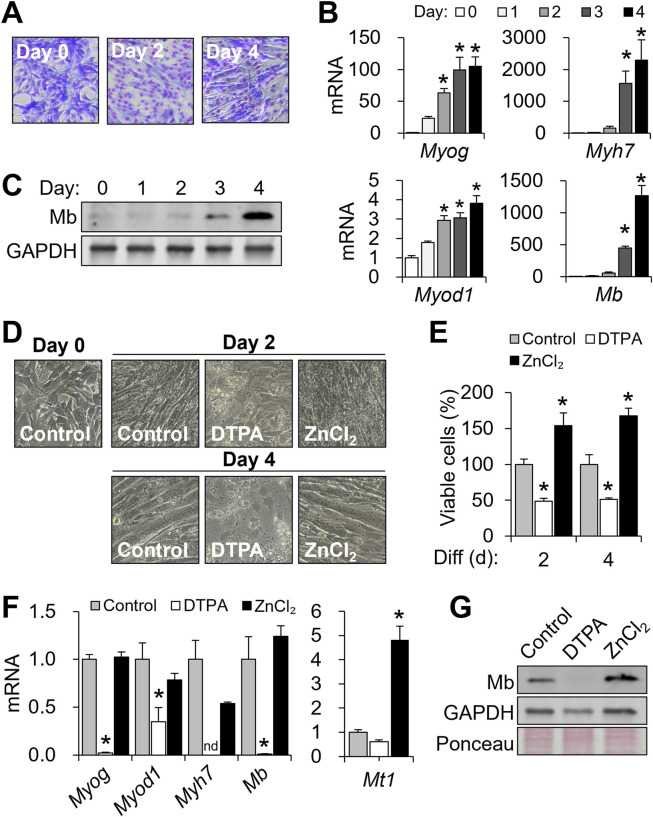
Cellular zinc depletion impairs C2C12 differentiation and myoglobin expression. To modulate cellular zinc status, C2C12 cells were treated with DTPA (50 µM) or ZnCl_2_ (50 µM) for 4 days during differentiation. **(A)** Myotube formation was visualized by Giemsa staining. **(B)** mRNA expression profiles of *Myog*, *Myod1*, *Myh7*, and *Mb* during differentiation. **(C)** Mb protein expression during myogenesis was examined by Western blot. **(D)** Representative images showing impaired myotube formation under prolonged zinc deficiency. **(E)** Viable cell counts assessed by CCK-8 assay. **(F)** Marked decreases in *Myog*, *Myod1*, *Myh7*, and *Mb* transcript abundance by prolonged zinc restriction (4 days). **(G)** Effects of zinc manipulation on Mb protein production shown with GAPDH and Ponceau S-stained total protein. Transcript abundance was normalized to *Actb* (n = 3, biological replicates). Values represent the mean ± SD. *P < 0.05. nd, not detected; d, days.

Upon initiation of differentiation, C2C12 myoblasts exit the cell cycle, activate myogenic transcription programs, and fuse into multinucleated myotubes (Tannu et al., 2004). Successful differentiation was confirmed by both morphological and molecular markers. Giemsa staining revealed the formation of multinucleated myotubes, indicating efficient fusion (Figure 1A). As expected (Panda et al., 2014; Zhang et al., 2020), transcription factor *Myog* and *Myod1* mRNA levels increased markedly during the 4-day differentiation period (Figure 1B). Expression of the muscle-specific gene *Myh7* and *Mb* also rose significantly, with protein abundance of Mb increasing notably between days 3 and 4 (Figures 1B,C).

To assess whether zinc availability influences myogenic progression, cells were treated with 50 μM DTPA or 50 μM ZnCl_2_ throughout differentiation. Zinc chelation by DTPA severely disrupted myotube formation, as indicated by altered morphology (Figure 1D), and significantly reduced cell viability (Figure 1E). In contrast, zinc supplementation enhanced counts of viable cells beyond the control levels, potentially reflecting increased proliferation (Figure 1E). At the molecular level, zinc deficiency suppressed the expression of *Myog*, *Myod1*, *Myh7*, and *Mb*, while zinc supplementation preserved their expression (Figure 1F). *Mt1*, a known zinc-buffering gene (Colvin et al., 2010), was significantly increased in response to zinc supplementation (Figure 1F). At a protein level, zinc deficiency not only reduced Mb expression but also decreased GAPDH levels, suggesting impaired overall cell metabolism (Figure 1G). In contrast, zinc supplementation substantially increased Mb expression (Figure 1G). Together, these findings highlight the vulnerability of myogenic differentiation and cell viability to zinc deficiency.

### 3.2 Zinc availability regulates *Mb* expression during C2C12 differentiation

Because prolonged zinc chelation with 50 μM DTPA for 4 days was cytotoxic, we used a milder condition (10 μM DTPA for 2 days) to evaluate the stage-specific effects of zinc deficiency. Cells were treated with 10 μM DTPA or 50 μM ZnCl_2_ during either the early (days 0–2) or late (days 2–4) stages of differentiation.

**FIGURE 2:**
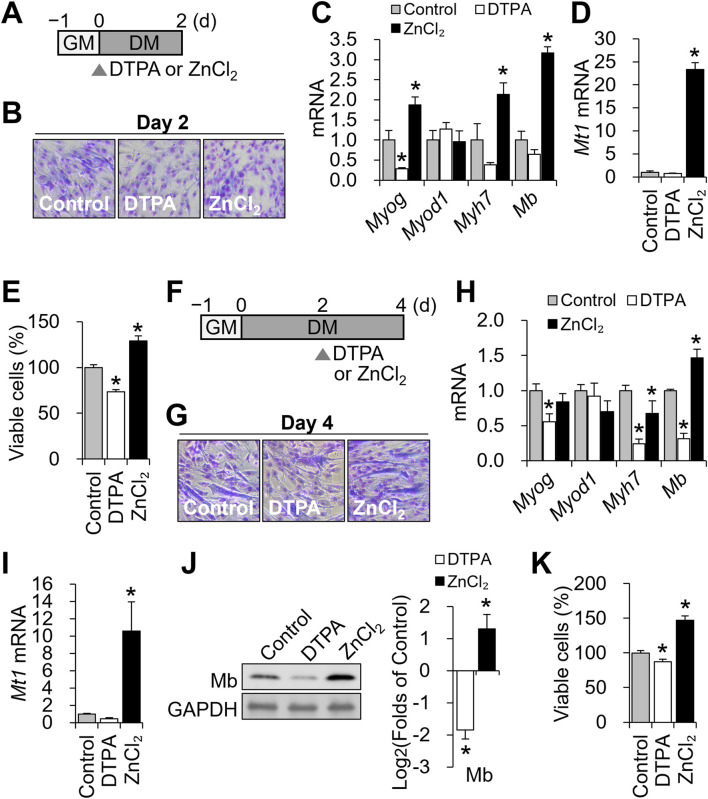
Zinc availability regulates Mb expression during distinct stages of C2C12 differentiation. **(A)** Schematic of early zinc manipulation for B-E. To modulate cellular zinc status during the early phase of differentiation, C2C12 cells were treated with DTPA (10 µM) or ZnCl_2_ (50 µM) for the first 2 days of the 4-day differentiation period. **(B)** Representative Giemsa-stained C2C12 cells at day 2 of differentiation under control, zinc-deficient, or zinc-supplemented conditions. **(C)** *Myog* mRNA expression was reduced under zinc deficiency, whereas zinc supplementation increased both *Myog*, *Myh7*, and *Mb* expression. *Myod1* expression remained unchanged regardless of zinc status. **(D)** Zinc supplementation markedly increased *Mt1* mRNA abundance. **(E)** Cell viability was reduced under zinc deficiency and enhanced by zinc supplementation, as determined by CCK-8 assay. **(F)** Schematic of zinc manipulation for G-K. To modulate cellular zinc status during the later phase of differentiation, C2C12 cells were treated with DTPA (10 µM) or ZnCl_2_ (50 µM) from day 2 to day 4 of the 4-day differentiation period. **(G)** Representative Giemsa-stained images of C2C12 cells at day 4 of differentiation under control, zinc-deficient, or zinc-supplemented conditions. **(H)** *Myog*, *Myh7*, and *Mb* mRNA expression were all reduced under zinc deficiency, while zinc supplementation increased *Mb* expression but decreased *Myh7* expression. *Myod1* expression remained unchanged regardless of zinc status. **(I)** Zinc supplementation significantly increased *Mt1* mRNA abundance. **(J)** Protein levels of Mb were reduced by zinc deficiency and increased by zinc supplementation. **(K)** Cell viability was reduced under zinc deficiency and enhanced by zinc supplementation. Transcript abundance was normalized to *Gapdh* (n = 3, biological replicates). Protein levels were normalized to GAPDH (n = 3, independent experiments). Values represent the mean ± SD. *P < 0.05. d, days; GM, growth medium; DM, differentiation medium.

We first examined the early phase, when myotube formation had not yet occurred (Figures 2A,B). At this stage, zinc status had already altered the expression of myogenic genes. *Myog* was significantly downregulated by zinc deficiency and upregulated by zinc supplementation, whereas *Myod1* remained unaffected (Figure 2C). *Myh7* and *Mb* expression was elevated with zinc supplementation but was not altered under zinc-deficient conditions, suggesting that zinc availability promotes the expression of these genes during early commitment. *Mt1*, a zinc-sensitive marker, was strongly upregulated by zinc supplementation, confirming cellular zinc sensing at this stage (Figure 2D). Counts of viable cells decreased by approximately 27% with 10 μM DTPA and increased with zinc supplementation (Figure 2E).

We next focused on the late differentiation phase (days 2–4; Figure 2F), during which myotube formation and *Mb* production become prominent. All groups formed multinucleated myotubes by day 4 (Figure 2G), indicating that zinc perturbation did not grossly impair fusion. As in the early phase, *Myod1* expression remained stable, while *Myog* was downregulated by zinc deficiency (Figure 2H). Notably, *Mb* transcript levels were significantly reduced under zinc-deficient conditions and elevated by supplementation, whereas *Myh7* expression decreased under both conditions, indicating differential zinc responsiveness during terminal differentiation. *Mt1* remained zinc-responsive with an increase by supplementation with ZnCl_2_ (Figure 2I). These changes were mirrored at the protein level: Mb was suppressed by zinc deficiency and enhanced by zinc supplementation (Figure 2J). Viability declined modestly (∼13%) with DTPA and improved with zinc supplementation (Figure 2K).

Together, these results demonstrate that zinc availability modulates *Mb* expression at both early and late stages of myogenesis, with stronger regulatory effects during the late phase when *Mb* expression and myotube formation are underway.

### 3.3 *Zip10* is the most zinc-responsive transporter during C2C12 differentiation

**FIGURE 3:**
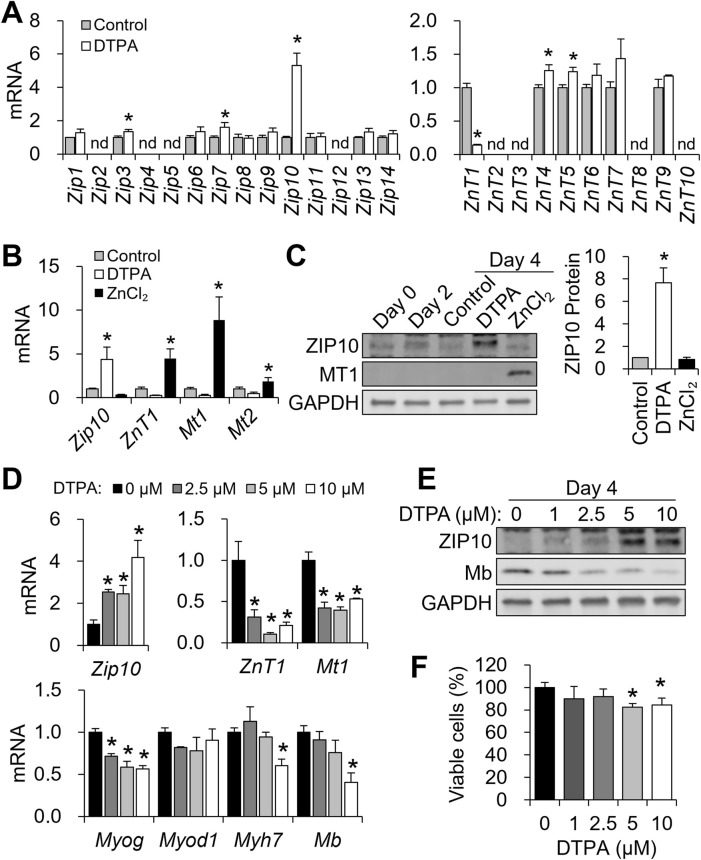
ZIP10 is highly responsive to zinc restriction during C2C12 differentiation. **(A–C)** To modulate cellular zinc status, C2C12 cells were treated with DTPA (10 µM) or ZnCl_2_ (50 µM) from day 2 to day 4 of differentiation. **(A)** Transcript levels of 14 *Zip* transporters and 10 *ZnT* transporters under zinc-deficient conditions during differentiation. **(B)** mRNA abundance of *Zip10*, *ZnT1*, *Mt1*, and *Mt2* was regulated in response to zinc status. **(C)** Western blot analysis of ZIP10 and MT1 expression in response to zinc status. Quantification of ZIP10 shows increased protein levels under zinc-deficient conditions. **(D–F)** Cells were exposed to increasing concentrations of DTPA (0–10 µM) from day 2 to day 4 of differentiation. **(D)** mRNA abundance of *Zip10* was dose-dependently increased by DTPA treatment, whereas *ZnT1* and *Mt1* expression levels were reduced under zinc-deficient conditions. In addition, *Myog* expression started to decline from 2.5 µM DTPA, *Myod1* remained unchanged, and *Myh7* and *Mb* expression was reduced only at 10 µM DTPA. **(E)** ZIP10 protein expression was markedly increased at 5 μM DTPA, and Mb protein levels decreased in a dose-dependent manner. **(F)** CCK-8 assay revealed a reduction in viable cells starting from 5 µM DTPA. Transcript abundance was normalized to *Gapdh* (n = 3, biological replicates). Protein levels were normalized to GAPDH (n = 3, independent experiments). Values represent the mean ± SD. *P < 0.05. nd, not detected; d, days.

To identify zinc transporters responsive to altered zinc status, we profiled all 14 *Zip* (*Slc39a*) and 10 *ZnT* (*Slc30a*) family members following 10 μM DTPA treatment from days 2–4 using qPCR. Among the *Zip* genes, *Zip3*, *Zip7*, and particularly *Zip10* were significantly upregulated under zinc-deficient conditions, with *Zip10* exhibiting more than a five-fold increase (Figure 3A). For *ZnT* transporters, *ZnT1* was downregulated, while *ZnT4* and *ZnT5* were modestly upregulated (Figure 3A). Further analysis revealed that zinc deficiency strongly induced *Zip10*, whereas zinc supplementation elevated *ZnT1*, *Mt1*, and *Mt2* (Figure 3B). At the protein level, ZIP10 abundance increased with DTPA, while MT1 rose under zinc supplementation (Figure 3C).

To examine dose-responsiveness, we applied graded DTPA (2.5–10 μM) during days 2–4. *Zip10* mRNA increased dose-dependently, with significant induction at 2.5 μM (Figure 3D). In contrast, *ZnT1* and *Mt1* expression declined at 2.5 μM DTPA and remained suppressed. *Myog* showed early sensitivity, with reductions from 2.5 μM DTPA, while *Myh7* and *Mb* expression decreased only at 10 μM. *Myod1* remained unaffected (Figure 3D). Protein expression mirrored transcript trends, where ZIP10 increased and Mb decreased with rising DTPA levels (Figure 3E). Cell viability remained stable up to 2.5 μM DTPA but declined significantly when ≥5 μM (Figure 3F). These results highlight *Zip10* as the most zinc-responsive transporter during myogenesis and establish that zinc deficiency suppresses key myogenic genes.

### 3.4 *Zip10* knockdown exacerbates Mb reduction under zinc-deficient conditions

**FIGURE 4:**
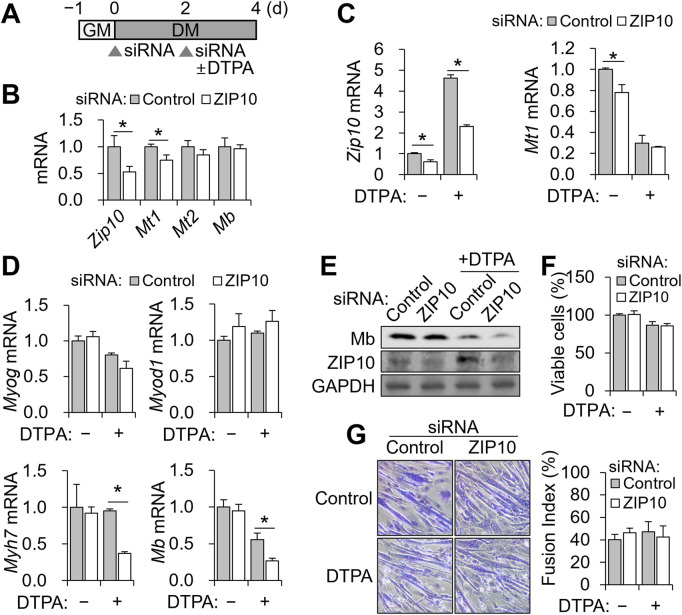
*Zip10* knockdown increases sensitivity to zinc deficiency during C2C12 differentiation. For mild zinc deficiency combined with *Zip10* knockdown, 5 µM DTPA was applied from day 2 to day 4 during differentiation. **(A)** Experimental scheme for combined treatment of DTPA and *Zip10* knockdown. **(B)** Relative mRNA expression of *Zip10*, *Mt1*, and *Mt2* following *Zip10* knockdown. Comparable expression of *Mb* following knockdown is shown. **(C)** Transcript abundance of *Zip10* validated the gene knockdown under zinc-depleted conditions. Relative mRNA expression of *Mt1* remained comparable under zinc-deficient conditions. **(D)** mRNA expression of *Myog* and *Myod1* remained unchanged, whereas *Myh7* and *Mb* expression was decreased following *Zip10* silencing under zinc-deficient conditions. **(E)** Western blot analysis indicating lower Mb synthesis by ZIP10 depletion under zinc deficiency. **(F)** Cell viability was not affected by *Zip10* knockdown, as assessed by CCK-8 assay. **(G)** Giemsa-stained images showing myotube morphology under *Zip10* knockdown and/or zinc-deficient conditions, with the fusion index remaining unaltered by *Zip10* knockdown. Transcript abundance was normalized to *Gapdh* (n = 3, biological replicates). Values represent the mean ± SD. *P < 0.05. GM, growth medium; DM, differentiation medium; d, days.

To test the functional relevance of *Zip10*, we performed siRNA-mediated knockdown during the 4-day differentiation, with 5 μM DTPA added from days 2–4 (Figure 4A). We chose 5 μM DTPA for zinc depletion, as this treatment markedly increased ZIP10 protein (Figure 3E). C2C12 cells were differentiated for 4 days, with siRNA transfected on days 0 and 2, and zinc deficiency induced from days 2–4 (Figure 4A). Knockdown efficiency was confirmed by reduced *Zip10* mRNA on day 4 (Figure 4B). In zinc-replete conditions, *Zip10* knockdown reduced *Mt1* expression, but *Mb* levels remained unchanged (Figure 4B).

Under zinc-deficient conditions, DTPA-induced *Zip10* upregulation was effectively blocked by siRNA (Figure 4C). However, *Mt1* expression was not further reduced (Figure 4C). Importantly, *Zip10* knockdown significantly decreased *Myh7* and *Mb* mRNA as well as Mb protein under zinc-deficient conditions, without altering *Myog* or *Myod1* expression (Figures 4D,E). Two-way ANOVA revealed significant interaction effects between zinc status and *Zip10* knockdown for *Myh7* and *Mb* expression (*P* < 0.05 for both), indicating that the impact of zinc deficiency on these genes depends on ZIP10 expression. In contrast, *Myog* and *Myod1* showed no significant interaction effects. Despite the decline in *Mb*, neither cell viability (Figure 4F) nor myotube morphology and fusion index (Figure 4G) was impaired. These findings demonstrate that ZIP10 is essential for sustaining *Mb* expression when zinc is limited, while overall differentiation and survival remain intact.

### 3.5 *Mb* expression is zinc-insensitive in mature myotubes

**FIGURE 5:**
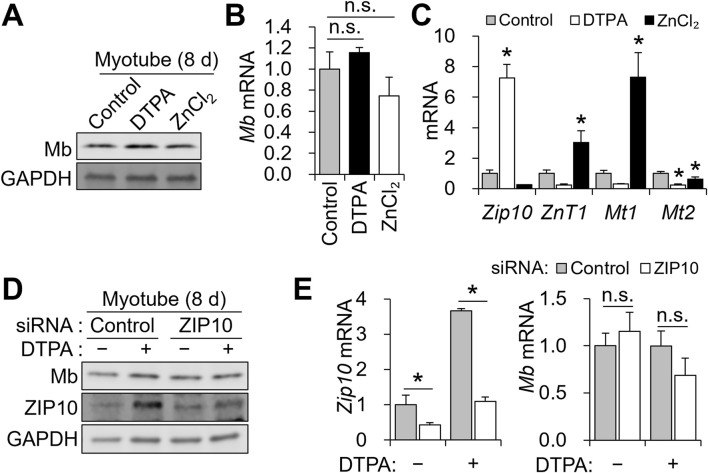
Mb expression is maintained in mature myotubes despite zinc restriction or ZIP10 deficiency. **(A–C)** C2C12 myoblasts were subjected to an 8-day differentiation, with 10 μM DTPA or 50 μM ZnCl_2_ applied from day 6 to day 8. **(A)** Protein expression of Mb remained unchanged by zinc manipulation. **(B)** mRNA abundance of *Mb* was comparable regardless of zinc status. **(C)** *Zip10* transcript abundance was upregulated under zinc deficiency. In contrast, zinc supplementation increased *ZnT1* and *Mt1* expression, while *Mt2* was consistently downregulated under both conditions. **(D–E)** For mild zinc deficiency combined with *Zip10* knockdown, 5 µM DTPA was applied from day 6 to day 8. **(D)** Mb protein expression was not reduced by ZIP10 deficiency in mature myotubes. **(E)** Transcript abundance of *Zip10* confirmed the gene knockdown under zinc-depleted conditions. Relative mRNA abundance of *Mb* remained comparable following *Zip10* silencing. Transcript abundance was normalized to *Gapdh* (n = 3, biological replicates). Values represent the mean ± SD. *P < 0.05. d, days.

Because zinc availability affected Mb expression in differentiating myoblasts, we next asked whether this regulation is maintained in mature myotubes. To address this, we treated C2C12 cells from days 6–8 with 10 μM DTPA or 50 μM ZnCl_2_ following 6 days of differentiation. In contrast to the clear reduction observed during the 4-day differentiation, *Mb* protein and transcript abundance remained largely unchanged upon zinc depletion or supplementation in mature myotubes (Figures 5A,B). Consistent with earlier observations, zinc deficiency continued to upregulate *Zip10*, while zinc supplementation enhanced *ZnT1* and *Mt1* expression. Notably, *Mt2* was suppressed under both conditions (Figure 5C). To further assess the contribution of *Zip10* to *Mb* regulation in mature myotubes, we performed siRNA-mediated knockdown of *Zip10* during days 6–8 of differentiation, in combination with 5 μM DTPA treatment. *Zip10* knockdown was again confirmed at both the protein (Figure 5D) and transcript levels (Figure 5E), validating the efficiency of siRNA transfection. However, *Mb* expression remained unchanged across all conditions (Figures 5D,E). These results indicate that *Mb* becomes zinc- and *Zip10*-independent once myotubes are fully matured.

### 3.6 Zinc restriction during myogenesis changes cellular NO metabolism

**FIGURE 6:**
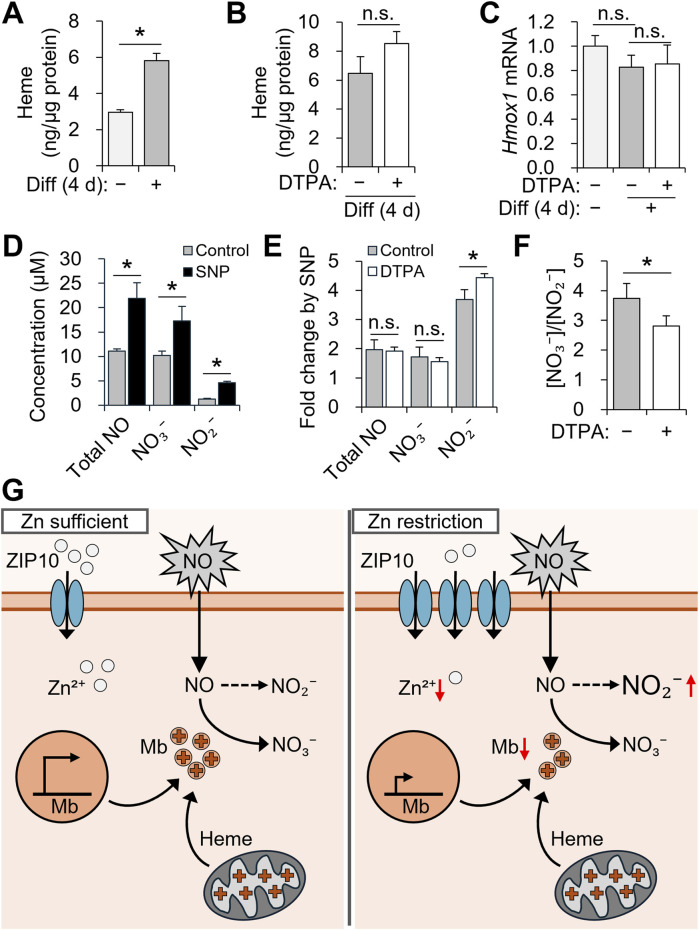
Zinc restriction during myogenesis alters nitric oxide metabolism in C2C12 cells. **(A–C)** C2C12 myoblasts were differentiated for 4 days with 10 µM DTPA applied from day 2 to day 4. **(D–F)** After differentiation, cells were exposed to 50 µM SNP for 6 h. **(A)** Cellular heme content was increased during myogenesis. **(B)** Cellular heme content was not significantly altered by zinc deficiency during differentiation. **(C)** The mRNA expression of *Hmox1*, which is involved in heme catabolism, remained unchanged under zinc depletion. **(D)** Total NO, NO_2_^−^, and NO_3_^−^ levels were increased following SNP treatment. **(E)** Zinc deficiency enhanced the SNP-induced increase in NO_2_^−^, while fold changes in total NO and NO_3_^−^ remained comparable. **(F)** The NO_3_^−^ to NO_2_^−^ ratio was decreased under zinc-deficient conditions, possibly due to reduced NO-scavenging capacity of Mb. **(G)** Schematic summary of the role of ZIP10 and Mb in NO metabolism during differentiation. Transcript abundance was normalized to *Gapdh* (n = 3, biological replicates). Values represent the mean ± SD. *P < 0.05. d, days; NO, nitric oxide; NO_2_^−^, nitrite; NO_3_^−^, nitrate; SNP, sodium nitroprusside.

Given that zinc deficiency reduced *Mb* expression during differentiation, we investigated downstream functional consequences, particularly in NO metabolism. *Mb* oxidizes NO to NO_3_^−^ under normoxic conditions (Kamga et al., 2012). Initially, we focused on heme production, since the heme moiety of Mb is essential for its interaction with NO (Kamga et al., 2012). As expected, total cellular heme increased after differentiation (Figure 6A). Notably, heme levels were unaffected by zinc depletion (Figure 6B), and *Hmox1* expression, encoding the rate-limiting enzyme in heme degradation (Choi and Alam, 1996), remained stable (Figure 6C).

To assess functional NO metabolism, C2C12 cells were differentiated for 4 days with zinc depletion from days 2–4 and then stimulated with 50 μM SNP for 6 h (Figures 6D–F). Compared to untreated controls, SNP treatment significantly increased the concentration of total NO, NO_3_^−^, and NO_2_^−^ in the culture supernatant (Figure 6D). While total NO and NO_3_^−^ levels were similar across groups, zinc-deficient myotubes exhibited significantly higher NO_2_^−^ (Figure 6E), resulting in a decreased NO_3_^−^/NO_2_^−^ ratio (Figure 6F). This shift suggests impaired conversion of NO to NO_3_^−^ under zinc-deficient conditions, likely due to reduced Mb abundance.

## 4 Discussion

Zinc is an essential trace element required for a broad spectrum of cellular processes, yet its specific role in skeletal muscle development and oxygen handling remains incompletely understood. Our study demonstrates that zinc deficiency impairs myogenic differentiation in C2C12 myoblasts, with ZIP10 upregulation acting as an adaptive mechanism to maintain intracellular zinc availability. Among the numerous zinc transporters expressed in muscle, ZIP10 (SLC39A10) emerged as particularly responsive to zinc depletion, consistent with previous reports identifying it as an MTF-1-regulated zinc importer that is activated under low-zinc conditions (Lichten et al., 2011). We observed that reduced zinc availability suppressed *Mb* expression during differentiation, while its expression was not affected by zinc in mature myotubes. These findings suggest that zinc and ZIP10 are most critical during the phases of myogenesis when Mb biosynthesis is highly active.

The zinc-responsive transcription factor MTF-1 is a conserved metal-binding regulator that controls the expression of metallothioneins and zinc transporters through interaction with metal response elements (MREs) in their promoters. Beyond its canonical role in maintaining metal homeostasis, recent mechanistic studies demonstrated that MTF-1 also participates directly in myogenesis (Tavera-Montañez et al., 2019). Upon initiation of myogenesis, MTF-1 expression and nuclear localization increase, and loss of MTF-1 impairs myotube formation. Importantly, MTF-1 binds to promoter regions of myogenic genes and forms a complex with MYOD1 protein, the master transcriptional regulator of the myogenic lineage, to promote their transcription. Although the previous work emphasized copper-dependent regulation, MTF-1 is also known to respond to zinc status via MRE binding and metallothionein gene activation (Balamurugan et al., 2004). Thus, it is plausible that zinc deficiency limits MTF-1-dependent activation of myogenic genes, thereby indirectly reducing *Myog* and *Mb* expression and delaying differentiation. This mechanism could explain the partial overlap between the effects of zinc restriction and ZIP10 deficiency, as both perturb intracellular zinc homeostasis and may converge on MTF-1 signaling.

While zinc is classically recognized as a cofactor for ALAD in the heme biosynthesis pathway (Jaffe, 2016), its impact on heme proteins beyond erythroid cells has been largely overlooked. We show that zinc deficiency markedly reduced *Mb* expression without measurably affecting total cellular heme levels, indicating that zinc affects the protein moiety of functional myoglobin rather than its heme prosthetic group. This suggests that zinc could influence *Mb* expression and stability through transcriptional and post-transcriptional mechanisms. Transcriptionally, zinc may act via zinc-finger transcription factors or epigenetic regulators. Transcription factors such as *Pw1/Peg3* and *Zfp422*, both containing zinc-finger domains, have been implicated in muscle development (Correra et al., 2018; Nie et al., 2020), while zinc-dependent enzymes such as histone deacetylases contribute to chromatin remodeling essential for myogenic gene expression (Porter and Christianson, 2019; Tian et al., 2020). Zinc limitation may impair recruitment or stability of such factors, thereby reducing transcriptional efficiency. At the post-transcriptional level, zinc-binding RNA-regulatory proteins including MBNL1/2 and tristetraprolin (TTP) modulate mRNA stability in muscle and other tissues (Konieczny et al., 2014; Brooks and Blackshear, 2013). Zinc deficiency could alter the structure or RNA-binding capacity of such proteins, thereby reducing *Mb* mRNA stability.

Functionally, suppression of Mb expression under zinc-limited conditions has potential consequences for skeletal muscle oxygen and NO metabolism. Myoglobin facilitates intracellular oxygen diffusion and scavenges NO, converting it to NO_3_^−^ to buffer redox status (Kamga et al., 2012). Genetic *Mb* knockout models exhibit preserved NO balance through compensatory angiogenesis and upregulation of alternative NO-handling enzymes (Godecke et al., 1999; Park et al., 2019). In contrast, the zinc deficiency model of our studies reduced the NO_3_^−^/NO_2_^−^ ratio without affecting total nitrate levels, indicating a distinct perturbation of NO homeostasis that may reflect incomplete compensation or simultaneous impairment of multiple NO-processing pathways. Zinc is also a structural component of endothelial nitric oxide synthase (eNOS) and plays an important role in general redox signaling (Ravi et al., 2004; Marreiro et al., 2017). Taken together, our findings suggest that zinc scarcity may simultaneously dampen both *Mb*-dependent and *Mb*-independent NO buffering, with potential consequences for mitochondrial function, vascular tone, and redox control in skeletal muscle.

These observations align with broader themes in zinc transporter biology and muscle pathophysiology. Loss-of-function mutations in ZIP13 lead to Ehlers-Danlos syndrome with connective tissue fragility (Fukada et al., 2008), while ZIP14-mediated zinc mislocalization has been implicated in muscle wasting and metabolic dysfunction in cancer cachexia (Wang et al., 2018). In addition, ZIP7 has been linked to glucose homeostasis and insulin signaling in muscle cells (Norouzi et al., 2019). Here, we show that ZIP10 upregulation partially preserves *Mb* expression during zinc deficiency, suggesting that functional ZIP10 activity may mitigate transcriptional stress under zinc-limited conditions. Genetic or epigenetic variation in ZIP10 could therefore influence individual susceptibility to muscle dysfunction in settings of elevated metabolic demand, inflammation, or subclinical zinc inadequacy. This hypothesis is consistent with emerging evidence that zinc homeostasis intersects with mitochondrial biogenesis, oxidative stress regulation, and insulin signaling, all of which are central to muscle health (Norouzi et al., 2019; Reddy et al., 2022).

Finally, these findings have translational relevance for cellular agriculture and alternative protein development. Cultured meat production faces ongoing challenges in recapitulating the appearance and flavor of conventional meat, primarily due to insufficient *Mb* accumulation in vitro-grown muscle fibers (Fraeye et al., 2020). Strategies such as hypoxic culture or iron supplementation have shown limited success or scalability (Fraeye et al., 2020). Our data demonstrate that zinc supplementation during differentiation robustly enhances *Mb* expression in C2C12 cells, supporting previous reports in livestock where dietary zinc improved meat redness (Saenmahayak et al., 2010; Liu et al., 2011). Zinc may act through multiple mechanisms, including activation of transcriptional programs and support of heme biosynthetic enzymes (Petrie et al., 1996; Kim et al., 2023). Moreover, given the responsiveness of ZIP10 to extracellular zinc, targeted manipulation of this transporter may further optimize intracellular zinc flux and *Mb* production. These findings highlight a previously underappreciated nutritional lever, zinc, for improving meat quality traits in engineered muscle systems.

In conclusion, our study identifies zinc and the zinc transporter ZIP10 as key regulators of *Mb* expression during early myogenic differentiation under zinc-limited conditions. We demonstrate that zinc availability modulates *Mb* transcription independently of heme levels, influences NO metabolism, and is partially buffered by ZIP10-mediated import. These findings expand the known roles of zinc in muscle gene regulation and suggest ZIP10 as a potential target for nutritional, genetic, or biotechnological interventions to support muscle function. In doing so, we bridge fundamental micronutrient biology with emerging applications in sustainable food technologies.

## Data Availability

The original contributions presented in the study are included in the article/Supplementary Material, further inquiries can be directed to the corresponding author.
