# Supplementary material for: Zinc transporter ZIP10 supports zinc homeostasis and myoglobin biosynthesis in differentiating C2C12 myoblasts
Source: Front Cell Dev Biol. 2025 Nov 19;13:1691317. doi: 10.3389/fcell.2025.1691317 (PMC12672441; doi:10.3389/fcell.2025.1691317)
Supplement: Supplementary file 1 [file Table1.docx]

Supplementary Material

**Supplementary Table 1**. Primer sequences for qPCR

| Gene of Interest | Primer Direction | Primer Sequence |
| --- | --- | --- |
| *Mt1* | Forward  Reverse | 5’ - CCTCCTGCAAGAAGAGCTGC - 3’  5’ - TTCGTCACATCAGGCACAGC - 3’ |
| *Mt2* | Forward  Reverse | 5’ - TGCGCTCGACCCAATACTCT - 3’  5’ - GCGGTTGAAGATCGACGAGA - 3’ |
| *Slc39a1 (Zip1)* | Forward  Reverse | 5’ - CCTGCATGTGACGCTTCAGT - 3’  5’ - CCAGCGTGATCTGCTCCATC - 3’ |
| *Slc39a2 (Zip2)* | Forward  Reverse | 5’ - TGGTTCTCACACTGGGCTGT - 3’  5’ - CTGTGGTGATGACCTGTAGCTG - 3’ |
| *Slc39a3 (Zip3)* | Forward  Reverse | 5’ - GCACCAAGCCTCAAGCTTCT - 3’  5’ - ACACAGGGCCAAAGCACAAA - 3’ |
| *Slc39a4 (Zip4)* | Forward  Reverse | 5’ - GCACAGCCACCCACTACATC - 3’  5’ - AGTCCCAGCACCTTGGGTAT - 3’ |
| *Slc39a5 (Zip5)* | Forward  Reverse | 5’ - TGACAGCCGTGTTTGCATCA - 3’  5’ - TGAAGCAGGGCAGACAGTACA - 3’ |
| *Slc39a6*  *(Zip6)* | Forward  Reverse | 5’ - TCTCTGCCCAGCCATCATCA - 3’  5’ - GCCACCAAGCCAGGCTATTT - 3’ |
| *Slc39a7*  *(Zip7)* | Forward  Reverse | 5’ - CGCATGCCTTGGAACCTCATT - 3’  5’ - CCACGAGGAAGGCGACAATC - 3’ |
| *Slc39a8*  *(Zip8)* | Forward  Reverse | 5’ - AGCTGCACTTCAACCAGTGTT - 3’  5’ - TCCTCGCAGGGATGGAAGTT - 3’ |
| *Slc39a9*  *(Zip9)* | Forward  Reverse | 5’ - TGTGCATTCCAGTGATGATCCA - 3’  5’ - CTGCAGCATGGACGACTAGC - 3’ |
| *Slc39a10 (Zip10)* | Forward  Reverse | 5’ - ACCGCCAGCATGAATGTTTGA - 3’  5’ - ATGCAGGGCAAAGGTACGTG - 3’ |
| *Slc39a11 (Zip11)* | Forward  Reverse | 5’ - GTGCCAGGAATCTGGCCATTG - 3’  5’ - CCGCTCAGCTGTCCATACCA - 3’ |
| *Slc39a12 (Zip12)* | Forward  Reverse | 5’ - TTGGAGAGGACTGGGTGTCC - 3’  5’ - GCTTCCAAACACAGCTTGCAG - 3’ |
| *Slc39a13 (Zip13)* | Forward  Reverse | 5’ - TACAGTCAGAAGCCGGAGCC - 3’  5’ - CCAGGAGTCCACCTAAGGCA - 3’ |
| *Slc39a14 (Zip14)* | Forward  Reverse | 5’ - CTCCATGTCTGTGCAGGACC - 3’  5’ - GCCAGTAGCAAGCACTCTGG - 3’ |

**Supplementary Table 1.** (Continued)

| *Slc30a1*  *(ZnT1)* | Forward  Reverse | 5’ - TCCGACTCCTTCCACATGCT - 3’  5’ - CCGAACGTGTTCTTCTGCGT - 3’ |
| --- | --- | --- |
| *Slc30a2*  *(ZnT2)* | Forward  Reverse | 5’ - TCGAGCCGAGATCCTTGGAG - 3’  5’ - GCAGCCCGAAGTGATCAACA - 3’ |
| *Slc30a3*  *(ZnT3)* | Forward  Reverse | 5’ - ACCCGTAAGGGACACCTTGT - 3’  5’ - AGCCGTGGAGTCAATAGCCA - 3’ |
| *Slc30a4*  *(ZnT4)* | Forward  Reverse | 5’ - GGCTTTCACAACGTTTCGCAT - 3’  5’ - AATGGCTTGGTACACCTTCCAG - 3’ |
| *Slc30a5*  *(ZnT5)* | Forward  Reverse | 5’ - TGCTGACACCAGTTTCCGTC - 3’  5’ - CATGGTGTGAATGGCCGTGA - 3’ |
| *Slc30a6*  *(ZnT6)* | Forward  Reverse | 5’ - TTTGGCTCATTGGCTGGGTC - 3’  5’ - TGTTCGTTCGCATCTCGTCG - 3’ |
| *Slc30a7*  *(ZnT7)* | Forward  Reverse | 5’ - ACTTCTAGAGGGACGGAGACC - 3’  5’ - GACAGGATGGACCTAAACCAGC - 3’ |
| *Slc30a8*  *(ZnT8)* | Forward  Reverse | 5’ - TATCGAGCAGAGATCCTCGGTG - 3’  5’ - GCTCACAGGCAAGGTACAGC - 3’ |
| *Slc30a9*  *(ZnT9)* | Forward  Reverse | 5’ - GTGCAGCGGCTTACTGAACT - 3’  5’ - GGATTGCCCTTACTGACGGG - 3’ |
| *Slc30a10*  *(ZnT10)* | Forward  Reverse | 5’ - ACATGGAAGAGCTGATGAGCCA - 3’  5’ - GCTGGCATCCTGGTATTCCG - 3’ |
| *Mb* | Forward  Reverse | 5’ - AGGAAGTCCTCATCGGTCTGT - 3’  5’ - ACGGTGCAACCATGCTTCTT - 3’ |
| *Myh7* | Forward  Reverse | 5’ - AAGCCTCAGCAGAGGAGTACA - 3’  5’ - ATGGCTGAGCCTTGGATTCTC - 3’ |
| *Myod1* | Forward  Reverse | 5’ - CTGCTCTGATGGCATGATGGA - 3’  5’ - ACTGTAGTAGGCGGTGTCGT - 3’ |
| *Myog* | Forward  Reverse | 5’ - TCCCAACCCAGGAGATCATTTG - 3’  5’ - GGCATGGTTTCGTCTGGGAA - 3’ |
| *Hmox1* | Forward  Reverse | 5’ - TGGTGCAAGATACTGCCCCT - 3’  5’ - GTCTGGGATGAGGTAGTGCTGAT- 3’ |
| *Gapdh* | Forward  Reverse | 5’ - CTCCACTCACGGCAAATTCAAC - 3’  5’ - GTAGACTCCACGACATACTCAGC - 3’ |
| *Actb* | Forward  Reverse | 5’ - AGGAGTACGATGAGTCCGGC - 3’  5’ - AGCTCAGTAACAGTCCGCCT - 3’ |
